# OsMSR3, a Small Heat Shock Protein, Confers Enhanced Tolerance to Copper Stress in *Arabidopsis thaliana*

**DOI:** 10.3390/ijms20236096

**Published:** 2019-12-03

**Authors:** Yanchun Cui, Manling Wang, Xuming Yin, Guoyun Xu, Shufeng Song, Mingjuan Li, Kai Liu, Xinjie Xia

**Affiliations:** 1 Key Laboratory of Agro-ecological Processes in Subtropical Region, Institute of Subtropical Agriculture, Chinese Academy of Sciences, Changsha 410125, China; mlwang@isa.ac.cn (M.W.); yxm@isa.ac.cn (X.Y.); limingjuan5002@163.com (M.L.); jxxia@isa.ac.cn (X.X.); 2 Zhengzhou Tobacco Research Institute of China National Tobacco Corporation, Zhengzhou 450001, China; gyxu@isa.ac.cn; 3 State Key Laboratory of Hybrid Rice, Hunan Hybrid Rice Research Centre, Changsha 410125, China; shufengsong@126.com

**Keywords:** *Arabidopsis*, small heat shock protein, *OsMSR3*, copper stress, reactive oxygen species

## Abstract

Copper is a mineral element essential for the normal growth and development of plants; however, excessive levels can severely affect plant growth and development. *Oryza sativa* L. multiple stress-responsive gene 3 *(OsMSR3)* is a small, low-molecular-weight heat shock protein (HSP) gene. A previous study has shown that *OsMSR3* expression improves the tolerance of *Arabidopsis* to cadmium stress. However, the role of *OsMSR3* in the Cu stress response of plants remains unclear, and, thus, this study aimed to elucidate this phenomenon in *Arabidopsis thaliana*, to further understand the role of small HSPs (sHSPs) in heavy metal resistance in plants. Under Cu stress, transgenic *A. thaliana* expressing *OsMSR3* showed higher tolerance to Cu, longer roots, higher survival rates, biomass, and relative water content, and accumulated more Cu, abscisic acid (ABA), hydrogen peroxide, chlorophyll, carotenoid, superoxide dismutase, and peroxidase than wild-type plants did. Moreover, *OsMSR3* expression in *A. thaliana* increased the expression of antioxidant-related and ABA-responsive genes. Collectively, our findings suggest that *OsMSR3* played an important role in regulating Cu tolerance in plants and improved their tolerance to Cu stress through enhanced activation of antioxidative defense mechanisms and positive regulation of ABA-responsive gene expression.

## 1. Introduction

Copper is an essential mineral element for the normal growth and development of plants. In plants, Cu functions as an important cofactor for metalloproteins and participates in numerous biological processes, including photosynthesis, respiration, oxygen superoxide scavenging, cell wall metabolism and lignification, and ethylene perception [1,2,3,4,5,6]. Cu-deficient soils not only affect the quality and quantity of plant food crops but also reduce their nutritional value as the main source of essential minerals for humans [7]. In addition, exposure of plants to excess Cu interferes with normal growth, proliferation, and differentiation of most plant cells [8,9,10,11,12,13,14]. One of the earliest and most obvious symptoms of Cu stress is inhibition of primary root elongation [15,16,17], while its prominent manifestations are decreased proliferation of root meristem cells [18], impaired cell integrity [19], and cell death [20]. Excessive accumulation of Cu in plants leads to the production of reactive oxygen species (ROS), which are toxic owing to their high redox activity [21]. 

Plants have developed specific mechanisms to prevent Cu toxicity by tightly regulating Cu homeostasis, including Cu uptake, translocation, efflux, and sequestration [22]. Plants also activate antioxidant defense responses to mitigate oxidative damage caused by free Cu ions in the cytosol [23]. The defense system includes ROS-removing enzymes, such as superoxide dismutase (SOD), peroxidase (POD), and catalase (CAT), as well as low-molecular-weight antioxidants, such as ascorbic acid (ASC) and glutathione (GSH) [24]. These antioxidant compounds and enzymes can be used as physiological indicators for evaluating plant antioxidant defense ability [15,16,17,18,19,20,21,22,23,24,25,26].

The stress hormone abscisic acid (ABA) plays an important role in plant stress tolerance. Heavy metals such as Cd, Hg, and Cu can induce the expression of ABA synthesis genes, which in turn increase the endogenous level of ABA [27,28]. A previous study showed that cadmium treatment increases endogenous ABA levels in cattail and reed roots [29], potato tubers [30], and rice plants [31]. Exposure to high Cu concentrations also increased ABA levels in rice [31]. During the germination of wheat seeds, ABA levels increased in the presence of Hg, Cd, and Cu stress [32]. Plants exposed to heavy metal stress showed an increased concentration of ABA, which indicates that the hormone is involved in the protective mechanism against heavy metal toxicity [27,33,34].

In plants, small heat shock proteins (sHSPs) with monomer sizes ranging from 12 to 42 kDa are more diverse and abundant than those in other organisms. There are 23 sHSPs in rice, and these are proposed to be categorized into fourteen classes [35]. Classes CI-CXI (nine subfamilies) are localized in the nucleus or cytoplasm, whereas the other five are positioned in the endoplasmic reticulum, mitochondria, plastid, and peroxisome [25,26,27,28,29,30,31,32,33,34,35,36]. These sHSPs are stimulated in response to a wide range of abiotic stresses. For instance, *Oshsp26*, which encodes a chloroplast-localized sHSP, has been shown to enhance tolerance against oxidative and heat stress in tall fescue [37]. Overexpression of sHSP17.7 enhances drought tolerance in transgenic rice [38]. Overexpression of *OsHsp18.0-CI*, an sHSP-CI family gene, enhances resistance to bacterial leaf streak in rice [39]. However, there are only a few studies on the sHSPs involved in heavy metal resistance.

Our previous studies have shown that OsMSR3 belongs to the class I sHSP family [40]. The expression of the *OsMSR3* gene significantly enhances tolerance of *Arabidopsis thaliana* (L.) Heynh (*A. thaliana*) to cadmium stress [41]. However, the molecular mechanism of *OsMSR3*-induced Cu tolerance is poorly understood. In this study, we determined that the expression of *OsMSR3* was upregulated by Cu stress. Therefore, we speculated that *OsMSR3* plays an important role in plant tolerance to Cu. Expression of *OsMSR3* enhanced the Cu stress tolerance of *A. thaliana*. Our research enhances the understanding of the role of sHSPs in heavy metal resistance in plants. 

## 2. Results

### 2.1. Expression of OsMSR3 is Induced by Cu Stress

**Figure 1:**
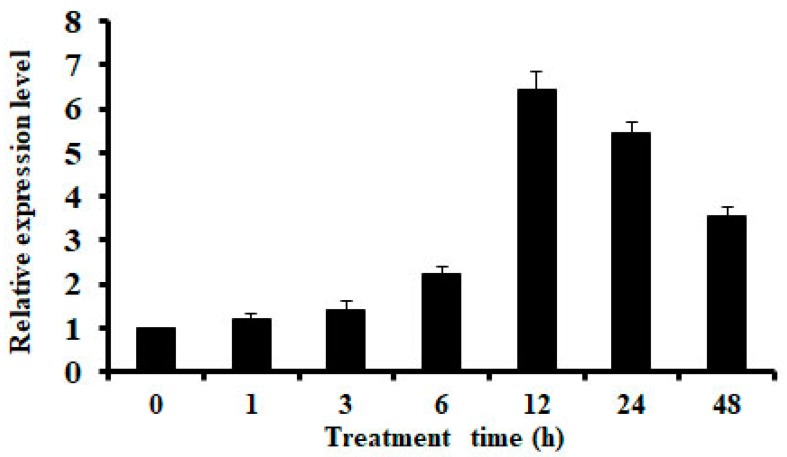
Expression analysis of the *Oryza sativa* L. multiple stress-responsive gene 3 (*OsMSR3*) gene. Expression analysis of *OsMSR3* in Pei’ai 64S rice seedlings at different time points (0, 1, 3, 6, 12, 24, and 48 h) under Cu stress using quantitative reverse transcription-polymerase reaction (qRT-PCR). The *ACTIN1* gene was used as an internal control. Error bars indicate standard deviations (SD) of three independent experiments.

The quantitative reverse transcription-polymerase chain reaction (qRT-PCR) showed that the expression of *OsMSR3* increased rapidly after 6 h of Cu stress and peaked at 12 h in Pei’ai 64S rice seedlings (Figure 1). Subsequently, *OsMSR3* expression decreased by nearly 3.5-fold compared to the control levels at 48 h (Figure 1). 

### 2.2. Expression of OsMSR3 Enhances Cu Tolerance of Transgenic A. thaliana

**Figure 2:**
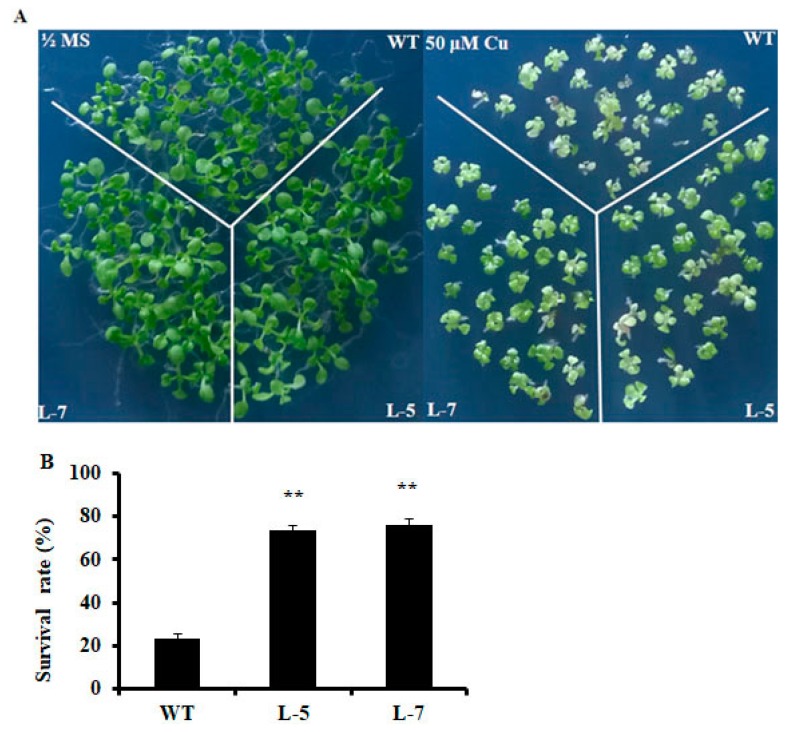
Performance of transgenic plants and wild-types under normal and Cu stress conditions. (**A**) Left panel, seedlings reared under normal conditions (0 μM CuCl_2_) for 30 days; right panel, seedlings exposed to 50 μM CuCl_2_ for 30 days. WT, wild-type *Arabidopsis thaliana*; L-5 and L-7, transgenic lines 5 and 7. (**B**) Survival rates of plants after growth under Cu stress for 30 days. Each column represents an average of three replicates, and bars indicate ± standard deviation (SD); and ** *p* < 0.01 indicate significant differences compared to wild-type T plants under the same conditions determined using Student’s *t-*tests.

**Figure 3:**
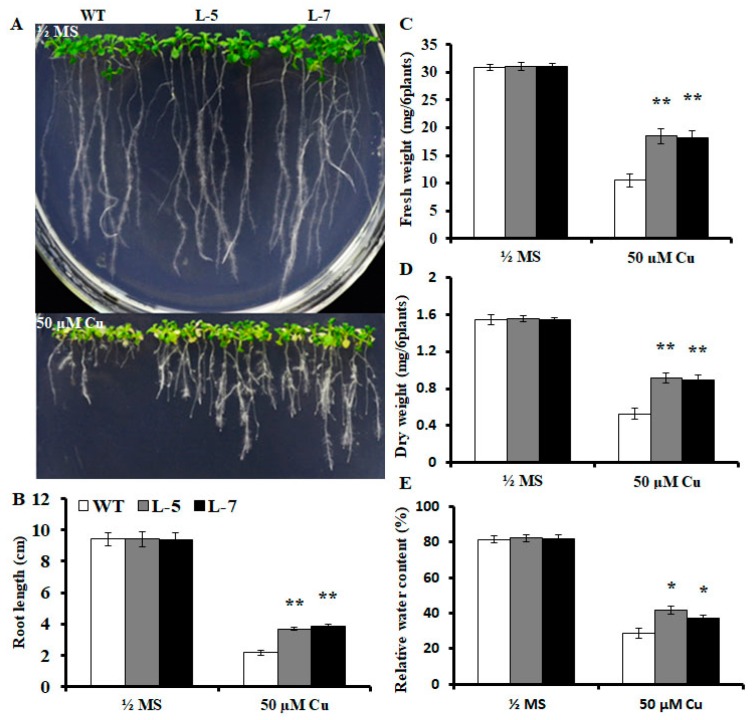
Improved tolerance to Cu stress induced by *Oryza sativa* L. multiple stress-responsive gene 3 (*OsMSR3*) expression. (**A**) Images of representative plants grown on half-strength Murashige and Skoog (½ MS) medium with or without 50 μM copper chloride (CuCl_2_) for 21 days. (**B**) Effect of Cu treatment on root length of plants presented in panel A. (**C**) Fresh weight (FW) and (**D**) dry weight (DW) of wild-type and transgenic line plants treated with or without 50 μM CuCl_2_ for 21 days. (**E**) Relative water content (RWC) of wild-type and transgenic plants treated with or without 50 μM CuCl_2_ for 21 days. Values are means ± standard deviation (SD) of three independent biological replicates; * *p* < 0.05 and ** *p* < 0.01 indicate significant differences from the wild-type determined using Student’s *t*-test.

To assess whether upregulation of *OsMSR3* enhances tolerance to Cu, transgenic *Arabidopsis* plants expressing *OsMSR3* were generated and analyzed. Based on a previous study [41], two independent transgenic lines, L-5 and L-7, were chosen for further experiments. We examined survival rates and root lengths of both *OsMSR3*-expressing and wild-type plants treated with Cu (50 μM). As shown in Figure 2A, there was no significant difference in survival rate and root length between wild-type and transgenic seedlings in the absence of stress. However, in half-strength Murashige and Skoog (½ MS) medium supplemented with Cu, transgenic plants showed higher Cu tolerance than wild-type plants did, with a higher survival rate and longer root length (Figure 2B and Figure 3B). Furthermore, the fresh and dry weight of wild-type and transgenic plants measured under normal and Cu stress did not differ significantly in the absence of stress, but fresh and dry weight were higher in both transgenic lines than they were in wild-type plants under Cu stress (Figure 3C,D). After the application of Cu stress, the relative water content (RWC) of transgenic plants was >35%. As shown in Figure 3E, under control conditions, the RWC was almost the same for all tested lines. In the presence of 50 μM copper chloride (CuCl_2_), all plants showed a reduction in RWC. However, water loss was higher in the wild-type than it was in the transgenic lines.

### 2.3. Expression of OsMSR3 in Arabidopsis Causes Higher Accumulation of Cu 

**Figure 4:**
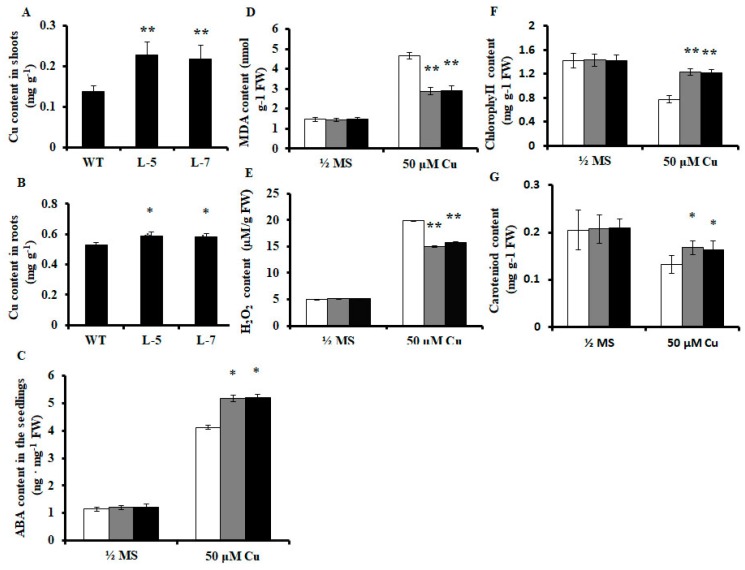
Quantitative analysis of various physiological indexes in wild-type and transgenic plants. (**A**,**B**) Cu content in wild-type and transgenic plant shoots and roots treated with 50 μM copper chloride (CuCl_2_) for 21 days, respectively. (**C**) abscisic acid (ABA), (**D**) malondialdehyde (MDA), and (**E**) hydrogen peroxide (H_2_O_2_) content in wild-type and transgenic plants treated with or without 50 μM CuCl_2_ for 24 h. (**F**,**G**) Chlorophyll and carotenoid content in wild-type and transgenic plants treated with or without 50 μM CuCl_2_ for 21 days. Values are means ± standard deviation (SD) of three independent biological replicates; * *p* < 0.05 and ** *p* < 0.01 indicate significant differences from wild-type plants under the same conditions determined using Student’s *t*-test.

To determine whether the enhanced Cu tolerance of transgenic plants was associated with their lower Cu accumulation, Cu content was determined in the different lines at the end of Cu treatment. Cu accumulation was higher in the roots and shoots of transgenic plants than that of wild-type plants. As shown in Figure 4A,B, the Cu content of transgenic lines L-5 and L-7 was approximately 1.2 and 1.1 times higher in the roots, and 1.66 and 1.59 times higher in the shoots, respectively, than it was in the wild-type plants. 

### 2.4. Effects of OsMSR3 Expression on ABA, Malondialdehyde (MDA), and Hydrogen Peroxide (H_2_O_2_) Content in A. thaliana

To determine whether OsMSR3 affects ABA content in *A. thaliana* under Cu stress, endogenous ABA content in transgenic and wild-type plants was measured. Under normal conditions, there was almost no difference in ABA content between the wild-type and transgenic lines, whereas Cu treatment increased the levels in both plant types (Figure 4C). Specifically, the mean ABA content, which was 4.12 ng g^−1^ fresh weight (FW) in wild-type plants, increased to 5.18 and 5.21 ng g^−1^ FW in the L-5 and L-7 lines, respectively (Figure 4C).

To examine the oxidative damage induced by excess Cu, we monitored the accumulation of malondialdehyde (MDA) and hydrogen peroxide (H_2_O_2_) in wild-type and transgenic plants. Under normal conditions, differences in MDA levels were not apparent between wild-type and transgenic plants, but levels were significantly increased by Cu stress (Figure 4D). Wild-type plants had a higher MDA content than transgenic plants did (Figure 4D). As shown in Figure 4D, MDA levels were approximately 1.61 and 1.59 times higher in wild-type plants than they were in transgenic lines L-5 and L-7 plants, respectively.

Cu stress can lead to H_2_O_2_ generation, which can be used to examine the oxidative damage induced by excess Cu [42]. In our study, H_2_O_2_ levels were not significantly different between transgenic and wild-type plants under controlled conditions. However, under Cu stress, H_2_O_2_ levels were lower in both transgenic lines than in wild-type plants, but no significant difference was observed between the L-5 and L-7 lines (Figure 4E).

### 2.5. Effects of OsMSR3 Expression in A. thaliana on Chlorophyll and Carotenoid Content

To determine whether the chlorophyll content is altered in transgenic plants under salt stress, we detected chlorophyll and carotenoid content in the leaves of wild-type and transgenic seedlings under normal conditions and Cu stress. As shown in Figure 4F,G, there was no significant difference in chlorophyll and carotenoid content between the transgenic and wild-type lines under normal growth conditions. In contrast, following Cu treatment, the chlorophyll content in the leaves of the *OsMSR3* transgenic lines (L-5 and L-7) was 1.59 and 1.57 times higher than that in the wild-type plants, although the content decreased in both transgenic and wild-type plants. The carotenoid content in L-5 and L-7 plants was 1.26 and 1.23 times higher than that in the wild-type.

### 2.6. Antioxidant Enzyme Activities are Altered in Transgenic A. thaliana

**Figure 5:**
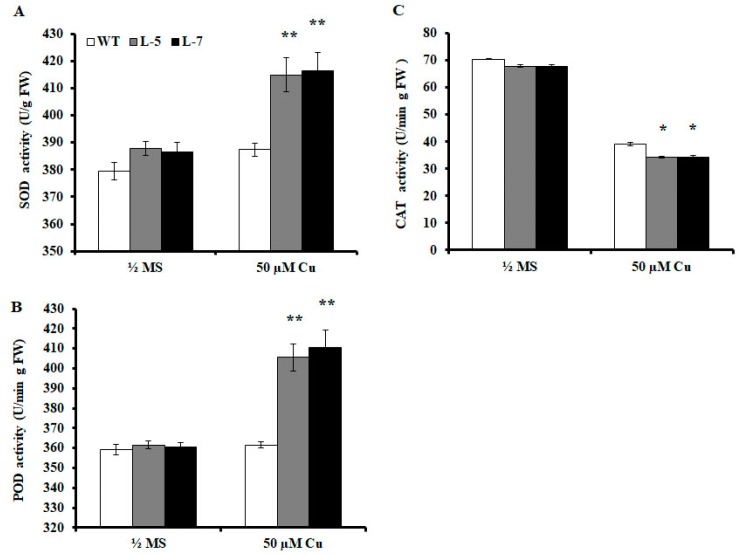
Antioxidant enzyme activity determination. Quantitative analysis of (**A**) superoxide dismutase (SOD), (**B**) peroxidase (POD), (**C**) and catalase (CAT) activity in wild-type and transgenic plants treated with or without 50 μM copper chloride (CuCl_2_) for 24 h. Values are means ± standard deviation (SD) of three independent experiments; * *p* < 0.05 and ** *p* < 0.01 indicate significant differences from wild-type plants under the same conditions determined using the Student’s *t*-test.

To determine whether increased Cu tolerance in transgenic plants is related to changes in oxidase activity in vivo, SOD, POD, and CAT activities were measured in wild-type and transgenic plants grown in medium without (CK) or with 50 μM CuCl_2_. The data showed that under normal conditions, SOD and POD activities in the transgenic lines were slightly higher than those in wild-type plants (Figure 5A,B), whereas CAT activity was slightly lower in the transgenic lines than in the wild-type plants (Figure 5C). Under Cu stress, SOD and POD activities increased in both wild-type and transgenic lines. However, the SOD activity of L-5 and L-7 was 1.07 times higher (Figure 5A), and the POD activity was 1.12 and 1.14 times higher (Figure 5B) than that of wild-type plants, respectively. Cu stress decreases CAT activity in both wild-type and transgenic lines with a greater decrease in the transgenic lines than in the wild-type plants (Figure 5C). The POD activity of the two transgenic lines was only 0.87 and 0.88 times higher than that of the wild-type plants (Figure 5C).

### 2.7. Expression of OsMSR3 Increases Expression of Antioxidant-Related and ABA-Responsive Genes

**Figure 6:**
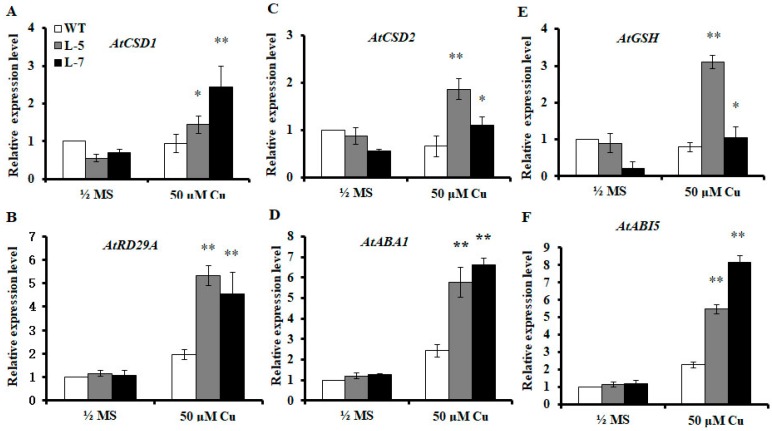
Quantitative reverse transcription-polymerase reaction (qRT-PCR) gene analysis. qRT-PCR analysis of relative expression of (**A**) *AtCSD1*, (**B**) *AtRD29A*, (**C**) *AtCSD2*, (**D**) *AtABA1*, (**E**) *AtGSH*, (**F**) and *AtABI5*, in two-week-old transgenic and wild-type (WT) plants treated with (Cu) or without (Control) 50 μM copper chloride (CuCl_2_) for 24 h. Values are means ± standard deviation (SD) of three independent biological replicates; * *p* < 0.05 and ** *p* < 0.01 indicate significant differences from wild-type plants under the same conditions, determined using the Student’s *t*-test.

To determine the performance of transgenic plants under Cu stress and elucidate the molecular mechanism underlying the resistance of transgenic plants to Cu stress, the transcript levels of antioxidant-related (*AtCSD1*, *AtCSD2*, and *AtPOD*) and ABA-responsive (*AtRD29A*, *AtABA1*, and *AtABI5)* genes were assayed in wild-type and transgenic plants under normal and stress conditions. The expression levels of *AtCSD1*, *AtCSD2*, and *AtPOD* were higher in wild-type plants than they were in transgenic plants under control conditions. However, higher gene expression was observed in transgenic plants than in wild-type plants under Cu stress conditions (Figure 6A–C). Compared to the expression under normal conditions, Cu stress inhibited the expression of these genes in wild-type plants but activated their expression in transgenic lines. The ABA-responsive genes, *AtRD29A*, *AtABA1*, and *AtABI5,* showed no significantly different expression levels between transgenic and wild-type plants under normal conditions (Figure 6D–F). Under Cu stress conditions, the expression levels of the three genes were higher in the transgenic lines than in the wild-type plants (Figure 6D–F). 

## 3. Discussion

Heavy metal pollution in soils is an emerging worldwide threat owing to its adverse effects on environmental safety [43]. Currently, cadmium and lead pollution in soils and their harmful effects on humans are attracting the attention of researchers globally. Cu pollution has become an important problem in the soil environment; however, studies of this phenomenon are still in infancy [44]. With the development of modern molecular biology, transgenic technology has emerged as an effective method to discover new Cu-tolerant genes in plants and cultivate plants that are highly efficient at repairing damage due to Cu contamination. In the previous study, we found that the expression of *OsMSR3* in *Arabidopsis* significantly enhanced tolerance to cadmium stress [41]. As Cu and cadmium belong to the group of heavy metal elements, we evaluated if the transgenic lines expressing *OsMSR3* in *Arabidopsis* could enhance the ability of copper tolerance.

The expression of *OsMSR3* was enhanced by Cu stress (Figure 1), which indicated that *OsMSR3* is involved in the response to Cu. Then, two transgenic lines L-5 and L-7 were used to perform the Cu tolerance experiment. The results showed that the expression of *OsMSR3* enhanced the tolerance of *A. thaliana* to Cu stress than that of wild-type plants, manifested as higher survival rate (Figure 2), higher biomass (Figure 3), and longer root length (Figure 3B). Moreover, transgenic plants accumulated less MDA than wild-type plants under Cu stress (Figure 4D). It is well known that Cu damages cell membranes by inducing lipid peroxidation [45]. The MDA level is used to detect membrane lipid peroxidation and permeability [46]. These results suggest that the expression of *OsMSR3* alleviated Cu-induced damage to the cell membrane of *A. thaliana*. Cell membrane stability is a major factor contributing to the maintenance of water status in plants during water deficit [47,48]. Therefore, the RWC of transgenic lines under Cu stress was higher than that of wild-type plants (Figure 3E). 

Although the transgenic plants accumulated more Cu in the root and shoot (Figure 4A,B), their growth was significantly better than that of the wild-type. The accumulation of Cu in plant roots may inhibit the development of fine roots and reduce the absorption of iron and other trace elements [49]. To some extent, OsMSR3 protein may reduce the inhibition of Cu transport from the root to the shoot. We suggest that OsMSR3 may be helpful in maintaining the homeostasis of Cu metal ions at the cell and plant levels. In addition, we found that chlorophyll and carotenoid content in the leaves of *OsMSR3* transgenic lines was higher than that in those of the wild-type plants under Cu stress (Figure 4F,G). Chlorophyll is an important part of the light-harvesting complex (LHCII). As an antenna for capturing light energy and transferring it to the reaction center, the chlorophyll content reflects the intensity of plant photosynthesis [50]. Carotenoids play an important role in plant growth and development. For example, they can act as a haptokine by transferring captured light to chlorophyll and can also act as a scavenger of free radicals in plant cells [51,52]. We speculated that the expression of *OsMSR3* reduced the damage to chlorophyll and carotenoids under Cu stress.

SOD activity and SOD-related gene expression in *OsMSR3* transgenic lines were significantly higher than those in wild-type plants (Figure 5A, Figure 6A,C). The presence of excess Cu causes the generation of ROS, such as superoxide radical (O_2_^−^), H_2_O_2_, singlet oxygen (^1^O_2_), and hydroxyl radicals (OH) [53]. To scavenge ROS and alleviate their deleterious effects, plants stimulate ROS-scavenging systems such as CAT, SOD, and POD [24], to combat the oxidative injury induced by heavy metal exposure [54]. SOD is the first line of defense against ROS and catalyzes O_2_^−^ to produce H_2_O_2_ and O_2_ [55]. An increase in SOD activity in stressed plants is an important indicator of superoxide ion production and enhancement of oxidative tolerance [55]. Therefore, the enhanced Cu tolerance of transgenic lines is related to the expression of SOD-related genes and SOD activity in *A. thaliana* under Cu stress induced by *OsMSR3* expression.

Cu stress can lead to the generation of ROS, such as H_2_O_2_ [56]. In this study, we found that H_2_O_2_ accumulated in wild-type and transgenic seedlings significantly during Cu stress, albeit to a lower extent in the two *OsMSR3* transgenic lines than in the wild-type (Figure 4E). The primary H_2_O_2_-scavenging enzymes in plant cells are CAT and POD; the former degrades H_2_O_2_ into water and oxygen. No studies, to date, have confirmed that a change in CAT activity is necessary to eliminate H_2_O_2_ in rice plants under Cu stress [55]. However, the current study revealed that Cu significantly increased POD activity in the transgenic lines but had little effect on CAT activity (Figure 5B,C). Moreover, *POD* gene expression was upregulated under Cu stress (Figure 6E). This is consistent with the increase in POD activity. Studies have shown that CAT has a high capacity but low affinity, whereas POD has a high affinity for H_2_O_2_ [57]. Thus, POD is the most effective H_2_O_2_-scavenging enzyme to reduce H_2_O_2_ content in plant cells under Cu stress.

Heavy metal exposure induces the expression of ABA synthesis-related genes in plants, which eventually leads to an increase in endogenous ABA levels [28]. In this study, under Cu stress conditions, ABA content and ABA-related gene expression levels in transgenic plants were significantly higher than those in wild-type plants (Figure 4C and Figure 6B,D,F). Therefore, we also propose that the expression of *OsMSR3* leads to the upregulation of ABA-related genes and an increase in endogenous ABA level under Cu stress, which may partly explain the increased tolerance of transgenic plants to Cu stress.

## 4. Materials and Methods

### 4.1. Plant Material and Growth Conditions

The seeds of rice (*Oryza sativa* ssp. *indica*) cultivar Pei’ai 64S were surface-sterilized with 75% ethanol for 2 min, treated with 50% sodium hypochlorite for 20 min, and then washed with distilled water at least thrice. The sterilized seeds were germinated on half-strength Murashige and Skoog (½ MS) medium and grown in a greenhouse under conditions of a light intensity of 600 μmol/m2/s 70% relative humidity, and 28 °C temperature with a 12-h light/dark photoperiod. For the Cu stress experiment, two-week-old seedlings were exposed to a nutrient solution containing 50 μM CuCl_2_ for 48 h. The leaves were harvested as a pool for each sample at 0, 1, 3, 6, 12, 24, 36, and 48 h after Cu treatment.

### 4.2. Cu Tolerance Assay

We used 50 µM and 100 µM CuCl_2_ to do the pre-experiment and then selected the concentration of 50 µM CuCl_2_ as the most suitable. The seeds of T3 transgenic and wild-type *A. thaliana* (ecotype Columbia-0) were surface-sterilized and sown in Petri dishes containing ½ MS media with or without 50 μM CuCl_2_. The seeds were incubated in the dark at 4 °C for two days to break the dormancy and then transferred to a growth chamber. After incubation for 30 days, the survival rate of *A. thaliana* was determined. For measurement of root growth under Cu treatment, three-day-old *A. thaliana* seedlings were transferred onto ½ MS medium with or without 50 μM CuCl_2_, in vertically placed dishes. After incubation for 21 days, the root length (from the base of the root to the tip) and FW of six plants were measured. 

Next, whole plants were rehydrated with distilled water at 4 °C for 12 h, blotted dry, and then the turgid weight (TW) was recorded. Rehydrated whole plants were oven-dried at 80 °C for 24 h, and the dry weight (DW) was recorded. RWC was calculated as follows: RWC (%) = (FW − DW)/(TW − DW) × 100. For the qRT-PCR analysis of selected genes, three-day-old *A. thaliana* seedlings were transferred onto ½ MS medium with or without 50 μM CuCl_2_. After 21 days of treatment, plant materials were harvested, and qRT-PCR was performed. The detailed procedure is provided in the next section. 

### 4.3. RNA Extraction and qRT-PCR Analysis

Total RNAs were extracted with TRIzol reagent (Invitrogen, Burlington, ON, Canada), as described previously [58]. qPCR analysis was conducted using AceQ qPCR SYBR Green Master Mix (Vazyme Biotech, Nanjing, China), and the reactions were performed using an ABI7900HT (Applied Biosystems, Foster City, CA, USA) and run on the following schedule: 95 °C for 10 min, followed by 40 cycles at 95 °C for 15 s and 58 °C for 30 s. The internal controls were *ACTIN1* and *β-TUBULIN* for rice and *A. thaliana*, respectively. The data for relative expression were analyzed using the comparative Ct method [59]. The primer pairs used in the qPCR analysis are listed in Table S1.

### 4.4. Measurement of Cu Content 

Cu content was determined according to the method described by Li et al. [24]. Briefly, three-day-old *A. thaliana* seedlings were transferred to ½ MS medium with or without 50 μM CuCl_2_. After 21 days of treatment, the roots and shoots were harvested and dried at 80 °C for two days. Dried plant tissues (50–100 mg roots; 100–200 mg shoots) were digested with 11 N HNO_3_ at 200 °C for 10 h. The digested samples were then diluted with 0.1 N HNO_3_ and analyzed using an atomic absorption spectrometer (Solaar M6; Thermo Fisher, Boston, MA, USA). The experiments were performed in triplicate.

### 4.5. Measurement of MDA Content

Two-week-old transgenic and wild-type plants were cultivated on ½ MS medium with or without 50 μM CuCl_2_ for 24 h. Then, 0.3 g of the seedlings was harvested and ground into a powder for the determination of MDA content, which was measured according to a previously standardized method [24].

### 4.6. Measurement of ABA Content

Two-week-old transgenic and wild-type plants were cultivated on ½ MS media with or without 50 μM CuCl_2_ for 24 h. Approximately 0.2 g of the leaf tissue was harvested, ground into a powder, and then suspended in 1.8 mL 100 mM sodium phosphate buffer (PBS, pH = 7.4) for ABA leaf content detection, using a previously published method [60].

### 4.7. Measurement of Chlorophylls and Carotenoids 

The chlorophyll and carotenoid content were determined according to a previous method [60]. Briefly, three-day-old *A. thaliana* seedlings were transferred onto ½ MS medium with or without 50 μM CuCl_2_ in vertically placed dishes. After incubation for 21 days, chlorophyll and carotenoids were extracted from the rosette leaves of the wild-type and transgenic plants with 100% alcohol. An ultraviolet-visible (UV-vis) spectrometer (UV-2600; Shimadzu Co., Kyoto, Japan) was used to measure the absorption of the extracts. The total chlorophyll and carotenoid content were calculated according to a previously published method [61].

### 4.8. Quantitative Analysis of H_2_O_2_

The H_2_O_2_ concentration was determined using a commercially available kit (Nanjing Jiancheng Bioengineering Institute, Nanjing, China). Briefly, two-week-old plants (wild-type and transgenic lines) cultivated in ½ MS medium were treated for 24 h with or without 50 μM CuCl_2_. Then, 0.5 g of the seedlings was harvested, weighed, immediately ground, and then suspended in 5 mL 0.9% sodium chloride solution. The supernatant was collected after centrifugation for 10 min at 4 °C and 3000× *g*, and the H_2_O_2_ content was measured according to the protocol provided by the manufacturer of the kit. 

### 4.9. Assay of Antioxidant Enzyme Activities

To measure antioxidant enzyme activities, two-week-old plants (wild-type and transgenic lines) cultivated in ½ MS medium were treated for 24 h with or without 50 μM CuCl_2_. Seedling samples (0.5 g) were frozen in liquid nitrogen, rapidly ground into powder, and then homogenized in 100 mM sodium phosphate buffer (pH 7.4) on ice. After centrifugation at 3000× *g* for 15 min at 4 °C, the supernatant samples were immediately used for the detection of antioxidant enzymes. The activities of SOD, POD, and CAT were measured using specific assay kits (A001-1, A084-3, and A007-1, respectively) from Nanjing Jiancheng Bioengineering Institute (Nanjing, China) according to the manufacturer’s instructions. 

### 4.10. Statistical Analysis

The experimental data were analyzed using the statistical package for the social sciences (SPSS) 17.0 statistical software (*SPSS* Inc., Chicago, IL, USA). At least three independent experiments were performed, and the average results are presented. Error bars represent standard deviation (SD, n > 3). Furthermore, * *p* < 0.05 or ** *p* < 0.01 indicate statistically significant means. 

## 5. Conclusions

In conclusion, we showed the involvement of *OsMSR3* in Cu tolerance in *A. thaliana*. *OsMSR3*-expressing lines exhibited enhanced Cu stress tolerance, possibly through enhanced activation of antioxidative defense mechanisms and positive regulation of ABA-responsive gene expression. In view of the good performance of the transgenic lines, *OsMSR3* can be used to modify plants for remediation of Cu pollution in the soil. Therefore, this study provides an important insight into plant biology and mechanisms to overcome increasing heavy metal pollution in soils.

## Supplementary Materials

Supplementary materials can be found at https://www.mdpi.com/1422-0067/20/23/6096/s1. Table S1. Primer sequences used for quantitative reverse transcription polymerase reaction (qRT-PCR).

## Abbreviations

ABA	abscisic acid
SOD	superoxide dismutase
POD	peroxidase
CAT	catalase
ROS	reactive oxygen species
ASC	ascorbic acid
GSH	glutathione
sHSPs	small heat shock protei
SD	standard deviation
