# Supplementary material for: OsMSR3, a Small Heat Shock Protein, Confers Enhanced Tolerance to Copper Stress in Arabidopsis thaliana
Source: Int J Mol Sci. 2019 Dec 3;20(23):6096. doi: 10.3390/ijms20236096 (PMC6929131; doi:10.3390/ijms20236096)
Supplement: Supplementary file 1 [file ijms-20-06096-s001.pdf]

Table S1. Primer sequences used for quantitative reverse transcription polymerase reaction (qRT-PCR)

Primer Name	Primer Sequence	Orientation
ACTIN1-F	ACAGGTATTGTGTTGGACTCTGG	Sense
ACTIN1-R	AGTAACCACGCTCCGTCAGG	Antisense
OsMSR3-F	GTCAAGTCCATCCAGGTTACCG	Sense
OsMSR3-R	GATCACAAACCGAAGGCTCG	Antisense
β-TUBULIN-F	GCTGACGTTTTCTGTATTCC	Sense
β-TUBULIN-R	AGGCTCTGTATTGCTGTGAT	Antisense
AtCSD1-F	TGATGGAACTGCCACCTTCACA	Sense
AtCSD1-R	ATGGCCTCCCTTTCCGAGGT	Antisense
AtCSD2-F	CCTCCTTCCTCCAATCCTTC	Sense
AtCSD2-R	AGCACTGCAACAGCCTTCTT	Antisense
AtPOD-F	ATGACTTACTACATGATGAGCTGTCC	Sense
AtPOD-R	CAGTGTTGTCTTTCGTTGAATCTAG	Antisense
AtRD29A-F	CAAACAGAGGAACCACCACTCAA	Sense
AtRD29A-R	CTGGTGCATCGATCACTTCAGGT	Antisense
AtABA1-F	CGTGCGGTTGGAGAAGATGTGAT	Sense
AtABA1-R	TCTCAGAATGGCTTCCTCCTCAGT	Antisense
AtABI5-F	GGGAAGGAAAAGAGTAGTGGAT	Sense
AtABI5-R	CCACTGTATATGCTTGTTTTCTT	Antisense
